# Maternal prenatal vitamin B12 intake is associated with speech development and mathematical abilities in childhood

**DOI:** 10.1016/j.nutres.2020.12.005

**Published:** 2021-02

**Authors:** Jean Golding, Steven Gregory, Rosie Clark, Yasmin Iles-Caven, Genette Ellis, Caroline M. Taylor, Joseph Hibbeln

**Affiliations:** a Centre for Academic Child Health, Population Health Sciences, Bristol Medical School, University of Bristol, Oakfield House, Oakfield Grove, Bristol, BS8 2BN, United Kingdom; b Department of Psychiatry, Barton Health, South Lake Tahoe, California, USA

**Keywords:** ALSPAC, Longitudinal cohort study, Pregnancy, Vitamin B12, Cognition, Speech, Mathematics, ALSPAC, Avon Longitudinal Study of Parents and Children, CCC, Children's Communication Checklist, CDI, MacArthur Communicative Development Inventory, DNA, Deoxyribonucleic acid, IQ, Intelligence quotient, NARA II, Neale Analysis of Reading Ability, SATS, Standardised Attainment Test Scores, tHcy level, Homocysteine, TOWRE, Test of Word Reading Efficiency, Vitamin B12, Cobalamin, WASI, Wechsler Abbreviated Scale of Intelligence, WISC, Wechsler Intelligence Scale for Children, WOLD, Wechsler Objective Language Dimension, WORD, Wechsler Objective Reading Dimension

## Abstract

Deficiencies of many nutrients in pregnancy have adverse effects on fetal brain development with consequent impaired cognitive function in childhood. However, it is unclear whether deficiencies of vitamin B12 prenatally are harmful to the developing fetus. We therefore used the Avon Longitudinal Study of Parents and Children to test the hypothesis that cognitive outcomes in childhood are reduced if their mothers consumed a diet low in vitamin B12 during pregnancy. A detailed exposome analysis was used to identify 9 factors independently associated with low vitamin B12 intake. These were taken into account in each of 26 outcome analyses. Results showed that the children of women with the lowest 10% intake of B12 were at increased risk of poor vocabulary at 24 months, reduced ability at combining words at 38 months, poor speech intelligibility at 6 years, poor mathematics comprehension at school years 4 and 6 (ages 8-9 and 10-11 years), and poor results on the national mathematics tests (age 13). There were no such significant adjusted associations for reading or spelling abilities, or for verbal or full-scale IQ (Intelligence Quotient) at 8 or at 15. Thus, we have confirmed that there are adverse effects on the child's development if the pregnant woman has a low intake of vitamin B12, and we have shown that these are specific to certain speech and mathematical abilities.

## 1 Introduction

There is substantial evidence to demonstrate that environmental exposures in pregnancy can have a major effect on the development of the child, particularly on cognition, since neurodevelopment occurs rapidly during the intrauterine period [1,2]. Diet in pregnancy, for example, has been shown to be particularly beneficial in regard to the child's intelligence quotient (IQ) if it contains fish [3,4]; iodine has also been shown to be one of the important components, with children born to women deficient in iodine having reductions in reading and verbal IQ [5].

In this study, we consider the mother's dietary exposure to vitamin B12 (cobalamin). This water-soluble vitamin is involved in the metabolism of every cell of the human body. It is involved in deoxyribonucleic acid (DNA) methylation through C1 metabolism and the homocysteine-methionine-S-adenosylmethionine pathway [6], axon myelination of neurons, and in the metabolism of both fatty acids and amino acids. Mouse models have even shown that epigenetic features such as DNA methylation can be altered by nutritional interventions and may thus change offspring phenotypes [7]. Consequently, it is likely to be particularly important in the normal functioning of the nervous system, and may have an important role in the growth and development of the fetal brain [8].

The development of the brain is more rapid in the early years of life compared to the rest of the body which could make it more vulnerable to nutritional deficiencies [7,9]. Further support for the importance of vitamin B12 in neurodevelopment comes from case studies of infants with deficiencies exhibiting cerebral atrophy and demyelination of nerve cells. After B12 treatment a rapid improvement in neurologic symptoms has been reported but many infants remained seriously delayed in development of cognition and language [7,10].

A review of the health effects of vitamin B12 by the World Health Organization [11] concluded that there was a need for considerable research into the possible adverse effects of vitamin B12 deficiency, but agreed that there was no clear definition of deficiency based on biological measures. Relating to general development, some studies have shown evidence of adverse effects identifiable at birth for children born to women who were deficient in the vitamin, including preterm delivery [12,13] and neural tube defects [11,^13^,14]. Studies of later development of children have shown inconsistent results. Some observational studies have shown an association between maternal vitamin B12 levels and offspring cognition (from 1 month to 10 years of age) including 2 from developing countries with high rates of B12 deficiency (India and Mexico) which found lower cognitive function in children whose mothers were deficient or had low (<2.0 µg/d) dietary intakes [1,^15^,16]. Identified were poorer levels of sustained attention and short-term memory in the Indian study and lower scores on the Bayley Mental Infant Development Measures in the Mexican study [1,^15^,16]. Two studies of an American cohort showed an association between maternal vitamin B12 dietary intake and offspring cognition (receptive language) at 3 years, but not at 7 years of age [1,^17^,18]. More recently a randomized controlled trial of oral B12 supplementation in India found no effect of prenatal maternal B12 supplementation on cognitive development of offspring at 9 months old, but elevated tHcy levels were associated with poorer cognitive performance [19]. Higher levels of tHcy are a biomarker for functional deficiency of different B vitamins such as B12, B6, and riboflavin and most closely linked to folate deficiency [19,20]. Possible reasons for lack of association in some studies could be due to the young age at assessment [19], or insufficient variation in B12 status in developed countries [21].

Here, we address the hypothesis that even in a developed country (the United Kingdom), low levels of vitamin B12 in the diet are associated with adverse cognitive development. We take advantage of data collected on the diet of more than 12,000 pregnant women in the Avon Longitudinal Study of Parents and Children (ALSPAC) on whom a variety of observational and scholastic tests have been undertaken throughout their childhood. In order to assess whether a low intake of vitamin B12 in utero is harmful to the brain of the developing child, we analyze the information on outcomes taking account of the features that are associated with a diet deficient in vitamin B12 in this population.

## 2 Methods and materials

### 2.1 Study sample

The study's pregnant population resided in the former county of Avon in south-west England. They were considered eligible if their expected date of delivery lay between the dates April 1, 1991 and December 31, 1992. Approximately 80% of the eligible population took part by completing questionnaires during, or shortly after, pregnancy. After delivery, the parents were followed up with self-completion questionnaires, detailed hands-on examinations of the children, tests designed for the study and administered in Avon schools, and linkage to the national school examination results of the child [22,23].

**Fig. 1:**
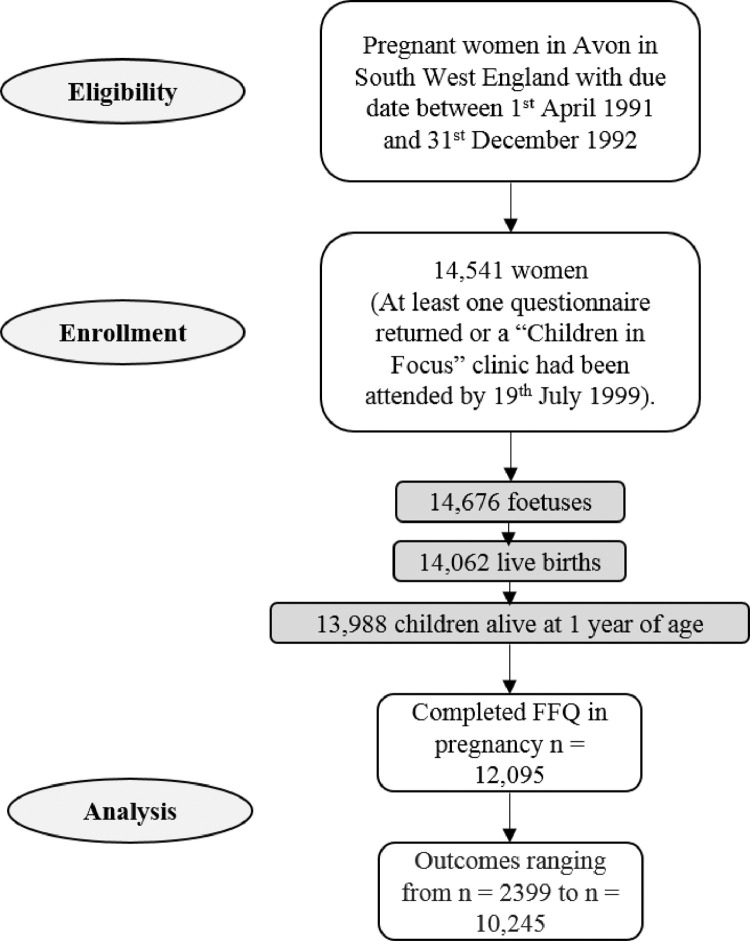
Number of participants included at each stage of the study.

The initial number of pregnancies enrolled was 14,541 (for these at least one questionnaire had been returned or a “Children in Focus” clinic had been attended by July 19, 1999). Of these initial pregnancies, there were a total of 14,676 fetuses, resulting in 14,062 live births and 13,988 children who were alive at 1 year of age (Fig. 1). The study website contains details of all the data that are available through a fully searchable data dictionary and variable search tool: http://www.bristol.ac.uk/alspac/researchers/our-data/.

Ethical approval for the study was obtained from the ALSPAC Ethics and Law Committee and the Local Research Ethics Committees that covered the study area. The ALSPAC Ethics and Law Advisory Committee agreed that consent was implied if questionnaires were returned [24]. For hands-on examinations consent was given by the parent and assent by the child.

### 2.2 Dietary intake

At 32 weeks gestation the mothers had received a questionnaire through the mail that requested a variety of information including the frequency with which they consumed a number of different foods and drinks nowadays. These covered the main foods consumed in the United Kingdom at that time, and comprised 43 food types each with five frequency options (never or rarely; once in 2 weeks; 1-3 times a week; 4-7 times a week; more than once a day). For 8 additional foods/drinks often consumed daily (e.g., coffee, tea, bread, and milk) more detailed questions were asked (see [25] for details).

Daily nutrient intakes were calculated from these questions using the nutrient content of foods as estimated in the fifth edition of McCance and Widdowson's “The Composition of Foods” and supplements (see [25] for details). Standard portion sizes used national data published in 1993 [26]. Calculated in this way the mean vitamin B12 daily intake was 4.87 µg/d (standard deviation 2.70), median 4.27 µg/d; we have taken below the 10th centile of the distribution of intake as the definition of a low intake for this set of analyses. In all, 10% of women consumed <2.264 µg/d (out of a total of 12,095 of the women enrolled in the study).

### 2.3 Outcomes

This study was designed to test the hypothesis that low intake of vitamin B12 during pregnancy would have an adverse effect on brain development, and that this would be demonstrated in the child's cognitive abilities. We had no prior hypotheses as to which cognitive abilities would be affected. We therefore tested all available measures for which there were considered sufficient numbers available. Details of all the questionnaires, tests and testing procedures are given on the ALSPAC website (see http://www.bristol.ac.uk/alspac/researchers/our-data/).

#### 2.3.1 IQ

##### 2.3.1.1 WISC scale

At 8 years of age the Wechsler Intelligence Scale for Children WISC-III ^UK^ [29] was used to assess cognitive function. Versions of this scale are the most widely used individual cognitive ability tests worldwide. A short form of the measure was employed in ALSPAC where alternate items (always starting with item number 1 in the standard form) were used for all subtests, with the exception of the coding subtest which was administered in its full form. Using this format, the length of the session was reduced and children were less likely to tire; such shortened forms of the WISC have been used successfully in several studies [30,31]. All tests were administered by members of the ALSPAC psychology team.

The final WISC IQ scores (verbal, performance, and total IQ) were calculated from the total subtest scores as described above. We used the scores traditionally used by researchers to assess IQ involving look-up tables. The mean (SD) of the total, verbal and performance subtests calculated in this way were: 104.0 (16.5); 107.0 (16.8); 99.5 (17.1), and each was normally distributed.

##### 2.3.1.2 Wechsler Abbreviated Scale of Intelligence (WASI)

The Wechsler Abbreviated Scale of Intelligence, Second Edition [32] is an updated abbreviated measure of cognitive intelligence designed for individuals 6 to 90 years of age. It was developed to quickly and accurately estimate cognitive intelligence when administration of a full battery is not feasible or necessary and was administered to the study offspring at age 15.

#### 2.3.2 Speech and language measures

##### 2.3.2.1 Vocabulary

Consistent with the developmental pattern of language acquisition, the analysis of children's expressive vocabulary in infancy was divided between an early phase (24 months of age) and a later phase (38 months). Expressive vocabulary scores were measured with age-specific-defined word lists (Anglicized for the Avon population) adapted from shortened forms of the MacArthur C*ommunicative Development Inventories* (CDI) and based on parent-report [33,34]. The measures have internal consistency, test-retest reliability and validity. A list of words was presented, each with possible responses: no does not say yet; understands but does not say; says and understands. These were scored 0, 1, 2 respectively and summed; thus, scores comprise both expressive and receptive language aspects. At age 15, the study children who attended the hands-on clinic were tested using the vocabulary test which was part of the WASI [35].

##### 2.3.2.2 Word combination and language scores

Questions were answered at 38 months in regard to the different ways in which the child combined words, used plurals and the past tense [36]. From these questions, 2 composite scores were derived—one referred to the combination of words, and the other, the language score, to the sum of the vocabulary, word combination, plurals, and past tense scores.

##### 2.3.2.3 Intelligibility

At 81 months (6 years), the child's mother was asked 3 questions concerning how often (1) the parents, (2) the family, and (3) visitors understood what the child said. Options were: never; sometimes; often; always, and were scored 0, 1, 2, and 3, respectively. The sum of these formed the intelligibility score.

##### 2.3.2.4 The comprehension score

The WOLD tests were used. According to the Technical Report for the WOLD, it was adapted for English participants from the scale produced by Wechsler in the United States. It was piloted on 4252 children in the United States and 800 in the United Kingdom by John Rust. The Anglicization of this process was undertaken by drawing on the combined expertise of educational psychologists, teachers, and researchers in the United Kingdom and aimed to make as few changes as possible from the US original. (The changes are listed in the Manual [36] and were carried out in 1994-5, thus having relevant vocabulary and norms for the 8-year-old ALSPAC children in 1999-2000.)

The WOLD included three subtests: listening comprehension, oral expression, and written expression. The reliability of the comprehension component as measured by split-half reliability ranged from 0.76 to 0.93, and the test-retest stability was 0.76.

##### 2.3.2.5 Pragmatic communication

At age 9 the study mother completed a questionnaire which included 7 of the 9 scales of the first version of the Children's Communication Checklist (CCC) [37]. This checklist was designed to assess aspects of communication that are not readily identified by conventional standardized tests including pragmatic aspects such as over-literal interpretation of stereotyped language. Although the CCC was initially designed to identify pragmatic difficulties, it has been shown to be good at discriminating a wide range of language and communication problems from typical development [38].

#### 2.3.3 Spelling

At both the 7- and the 9-year clinics, a spelling test was administered immediately after the reading session. A total of 15 words were chosen specifically for each age group after piloting on several hundred children by Peter Bryant and Terezinha Nunes of the Department of Education at the University of Oxford. The spellings involved regular and irregular words of different frequencies. They were given in order of increasing difficulty as identified from the pilot studies. For each, the word was read out aloud to the child, and then within a specific sentence incorporating the word, and then alone again. The child was asked to write down the spelling even if he/she thought they were just guessing at the spelling. The spelling score was the number of words spelt correctly (range 0-15).

The spelling scores for each age included those who stopped early (usually because they had reached the limit of their ability) [39]. The scores were not normally distributed but did not have ceiling effects. Scores were available for 8031 and 7640 of the 7- and 9-year-old children, respectively.

#### 2.3.4 Reading

##### 2.3.4.1 Word reading

For the reading assessment at age 7, the basic reading subtest of the WORD (Wechsler Objective Reading Dimension) was used for all children attending the ALSPAC clinic. Pictures and words were used to assess decoding and word reading [40].

The child was shown a series of four pictures by trained psychologists. Each picture had 4 short simple words underneath it. The child was asked to point to the word which had the same beginning or ending sound as the picture. This was then followed by a series of 3 pictures, each with 4 words beneath, each starting with the same letter. The child was asked to point to the word that correctly named the picture. Finally, the child was asked to read aloud a series of 48 unconnected words which increased in difficulty. If the child read the word incorrectly but pronounced it in a way that was phonetically plausible, this was also noted for each word. The reading task was stopped after the child had made six consecutive errors. 8070 children completed the task; the score was the number of items the child responded to correctly. It ranged from 0 to 54, with mean 28.1 (SD 9.44).

At age 9 years, the child was asked to read aloud 10 real words, followed by 10 nonwords. Both the words and nonwords were selected from a larger selection of words taken from previous research [41]. The 2 sets of words were specifically chosen for this study by Nunes and Bryant.

The test-retest reliability of the word reading is 0.8, and the scale has a correlation of 0.85 with the Schonell Word Reading Task [42]. Under test conditions, the child was shown each word in turn and asked to read the words out loud. The word reading score was calculated as the number of words read correctly (range 0-10). A total of 7657 children had a score which ranged from 0 to 10, but the distribution demonstrated a ceiling effect.

##### 2.3.4.2 Reading comprehension at 9

The revised Neale Analysis of Reading Ability (NARA II) [43] was used to assess the child's reading skills and comprehension at age 9 years. This test is suitable for children between the ages of 6 and 12 years with a standard assessment time of 20 minutes. It was administered by trained psychologists using Form II. The testing took place in a quiet room. Wherever possible, parents were asked not to accompany their child into the testing room to minimize distractions and interruptions.

The child was first given a practice story; the same structure for testing was used for this and all subsequent test passages. A booklet was used from which each child read a passage. They were then asked a series of questions about the content of the story they had just read. The tester recorded the time (in seconds) it took the child to read the passage. Any errors made by the child during reading were noted on the data sheet. The child was prompted by the tester if they: (1) mispronounced a word; (2) substituted a word; (3) refused to say a word; (4) made an addition (only if it altered the meaning of the story); (5) made an omission; or (6) reversed a word.

Administration of the test was undertaken following the instruction manual. If the child made more than 17 errors on the practice passage, the tester did not ask the child the comprehension questions but moved straight on to the level 1 story. All other children moved on to the level 2 story unless the tester felt that they had difficulty with reading the practice passage. If the child made less than 3 errors on the level 2 story, the tester moved on to level 3. If, however, the child made 3 or more errors on level 2, the comprehension questions were administered but the tester moved down to the level 1 story (only moving on to level 3 if the child completed level 1 within the permissible number of errors). For the remaining test passages, the child was not asked the comprehension questions if they made more than 16 errors (20 on level 6) and the session was ended.

The comprehension questions were asked as soon as the child had finished reading. For each question, the child was given 10 to 12 seconds to respond; they could refer to the text to assist them.

The raw comprehension score was obtained by summing the number of correct answers the child gave for each passage. If the permissible number of errors was exceeded for the final passage, the comprehension questions were not asked so no score was given for that passage. The conversion of the raw score to a score standardized for age used the test authors’ criteria. It was approximately normally distributed and available for 6943 children. It should be noted that 48 children were unable to attempt the test and have been excluded. However, when examining the outcome of reading impairment, we have included these 48 children in the impaired group.

##### 2.3.4.3 Reading speed

Using the times taken for the child to read each passage, a speed rate of words per minute was computed for each child. This was based on only those passages read where no more than 16 errors were made (20 for passage 6) and was created as follows:

Rateperminute=Totalno.wordsread×60/Totaltimetaken(sec)

The reading speed standardized for age was approximately normally distributed with mean 105.1 (SD 12.6).

##### 2.3.4.4 Reading fluency or sight-word efficiency

At the 13-year assessments, a word reading task (the TOWRE task) was given to the study children. It provides a test of sight-word efficiency [44]. A list of 104 words was given to the child to read, and the number read accurately within 45 seconds was recorded. The score identifying the number of words read in the time (but not the accuracy) was approximately normally distributed.

##### 2.3.4.5 National tests of reading proficiency

The study linked to the offspring's national test results known as SATS. These 14-year-old assessments were administered at a mean age of 14 years 1 month. The reading test comprised: (1) Reading a paper and answering questions (1hour 15 minutes) (Max 32 marks); (2) reading a piece of Shakespeare and answering questions on its content (45 minutes) (Max 18 marks). All children were eligible for these tests if they lived in England or Wales.

#### 2.3.6 Mathematics and science

The development of numeracy and mathematical skills is made up of several components which are built on in hierarchical ways over time [45]. Even before children enter formal schooling, they intuitively start to piece together basic mathematical concepts such as relative size and counting. Much of the research on the development of mathematical skills has focused on arithmetic or word problem solving but little is known about influences on the general course of mathematics performance in nonselected populations.

##### 2.3.6.1 Mathematical reasoning tests

To obtain data appropriate to the aims of ALSPAC, various advisors were asked for their opinions over time. These included expert researchers including Terezhina Nunes and Peter Bryant, as well as representatives of the local Avon education authorities and other expert contributors. The result was the recommendation to use tests devised for the study by Nunes and Bryant which would ensure the measurement of mathematical reasoning. The aim of these Mathematical Reasoning tasks was to assess children's understanding and use of quantitative relations to solve mathematical problems. They designed two different Mathematical Reasoning tasks. One, containing 17 items, was given to schoolchildren in year 4 (N = 5275, mean age 8 years 9 months). The other, containing 35 items, was given to children in year 6 (N = 7981, mean age 11 years 2 months) and again in year 8 (N = 2755, mean age 12 years 8 months).

The aim of these tasks was to assess children's reasoning about quantities and the relations between quantities in mathematical problems independently of their computation skills. None of the items in these tests contained difficult calculations; the children had to reflect on the relations between quantities in each item to decide how to solve the problem. All the items were presented with the support of drawings; the children could use counting to solve many of the problems if they did not know the number facts that might be used in the solution. All the problems were presented orally by the teachers to avoid an undue influence of reading difficulties on the children's performance [46].

Three types of item were included in the year 4 Mathematics Reasoning Task: additive reasoning items about quantities, additive reasoning items about relations, and multiplicative reasoning items about quantities. The assessments used in Years 6 and 8 included 6 types of item: additive reasoning items about quantities; additive reasoning items about relations; multiplicative reasoning items about quantities; multiplicative reasoning items involving relations (i.e., proportions); items about spatial reasoning and items about fractional quantities. Analyses of their internal consistency using Cronbach's α showed that on all 3 occasions the mathematics reasoning tasks had good levels of interitem reliability: 0.74 at year 4 (N = 5275), 0.89 at year 6 (N = 7881), and 0.91 at year 8 (N = 2755). This high internal consistency justifies the addition of all the items in each school year into single scores.

##### 2.3.6.2 Mental arithmetic

Arithmetical ability was measured as part of the WISC verbal intelligence tests at age 8 years. The raw scores at age 8 years were measured using alternate questions as for the WISC test in general [47]. The data were approximately normally distributed.

##### 2.3.6.3 National tests of mathematics

The results of the school-administered Standardised Attainment Test Scores (SATS) at age 10 to 11 years (known as “key stage 2,” KS2) and at 13 to 14 years (known as “key stage 3,” KS3) were obtained from the UK government (Department of Children, Schools & Families). SATS tests are scored in each subject at levels 1 to 8. The UK government recommendations are that children should achieve a level 4 by KS2 and a level 5 by KS3 and that they should progress by at least 1.0 unit or level every 2 years [48]. Thus, a coefficient of 0.5 represents an average 1 year of progress.

##### 2.3.6.4 Scientific reasoning

A test of scientific reasoning was developed specifically for the study by Nunes and Bryant. The aim was to measure children's understanding that in a properly controlled scientific comparison, one variable is tested at a time while other variables are held constant. The test was administered in school year 6 (age 11-12 years) by the class teacher. The pupils’ scores successfully predicted their later progress in science even after allowance was made for age and IQ [49].

### 2.4 Choice of potential confounders using the exposome technique

ALSPAC collected a considerable amount of data on the parents and grandparents before the child was born. To determine the factors that were associated with a low maternal vitamin B12 intake, we carried out an exposome analysis, using all data in the study excluding those which might have been on the causal pathway. Consequently, we did not consider factors such as maternal depression or the child's birthweight which could have been a consequence of the mother's deficiency. As in previous ALSPAC studies using the exposome methodology [27,28], we considered the variables in groups and ascertained those variables that were associated with low vitamin B12 intake at *P* < .05.

### 2.5 Statistical analyses

Step-wise logistic regression was then undertaken in each group to determine the intragroup independent factors. The groups were combined, and further stepwise logistic regressions were then undertaken with combinations of models until a final model was identified (see Supplemental Tables). The variables in the final exposome model were then used as confounders in the analyses of the associations between prenatal vitamin B12 intake and child outcomes. P<0.05 was considered statistically significant.

## 3 Results

### 3.1 Identification of confounders

The potential confounders used are delineated in Supplemental Tables S1 to S5. In all, 28 variables had unadjusted associations with low vitamin B12, mostly at *P* < .0001. These 28 variables were divided into three groups: (1) mother in childhood; (2) demographic features at time of pregnancy, and (3) lifestyle during pregnancy. The backward stepwise analysis of features of her childhood kept 7 of the 12 items in the analysis; the demographic analysis kept 3 of the 10, and the lifestyle retained all items except passive smoke exposure. Combining the items retained in each group into a final stepwise regression resulted in 9 items being retained (Supplemental Table S5). The 9 variables independently associated with low intake were: mother being nonwhite; having poor care from her own mother during childhood; her age (the older the less likely to have a low intake); her social network (the fewer helpful contacts the more likely to have a low intake); her education level (those who had achieved more qualifications were less likely to have a low intake); parity (the mothers who had had previous births were less likely to have a low intake); smoking in pregnancy at 18 and 32 weeks gestation (the more cigarettes smoked the more likely the diet to be low in vitamin B12); and the mother's locus of control (the more externally oriented she was, the more likely to have a diet low in vitamin B12). These 9 variables were all taken into account when assessing the child's cognitive outcomes.

### 3.2 Identification of outcomes

A total of 28 outcome variables were selected (see Section 2.5) to represent the cognitive aspects of the child's development in the areas of (1) IQ levels; (2) speech and language; (3) mathematics and scientific understanding; and (4) reading and spelling abilities. We had restricted the variables under consideration to those where the outcomes were measured on a continuous scale and numbers of cases available were in excess of 4000. We made one exception to this rule post hoc: having found adjusted associations with mathematics comprehension in school years 4 and 6, we decided to include mathematics comprehension in school year 8 even although there were only 2399 child results available.

#### 3.2.1 IQ

Before adjustment, the tests of IQ at 8 and at 15 years showed strong associations with low vitamin B12 level, but after adjustment the only association remaining with a statistically significant association at *P* < .10 was a weak association with Performance IQ (b = −1.29; 95% confidence interval [CI]−2.77, +0.20; *P* = .090; Table 1).

**Table 1:** Intelligence test outcomes in children

Test	Unadjusted			Adjusted		
	n	b [95% CI]	*P*	n	b [95% CI]	*P*
Verbal IQ at 8 years	6667	**−3.19 [−4.63, −1.75]**	**<.0001**	6334	−0.50 [−1.88, +0.89]	.481
Performance IQ at 8 years	6657	**−2.87 [−4.34, −1.39]**	**<.001**	6324	**−1.29 [−2.77, +0.20]**	**.090**
Total IQ at 8 years	6637	**−3.34 [−4.76, −1.92]**	**<.0001**	6305	−0.83 [−2.19, +0.54]	.237
Total IQ at 15 years	4778	**−7.07 [−10.01, −4.12]**	**<.0001**	4281	−0.27 [−1.57, +1.03]	.685

The unadjusted and adjusted mean differences (b) between the tests of IQ for children of mothers consuming <2.264 µg/d compared to children whose mothers consumed >2.263 µg/d of vitamin B12 during pregnancy. Values are mean IQ points with 95% confidence intervals. Adjustments made for: mother's ethnic background; her care by her mother in childhood; her age; her current social network; her level of educational attainment; her parity 0 vs > 0 (i.e., child has older siblings); her locus of control (external vs internal), and smoking habits at 18 and 32 weeks. Results with *P* values <.10 are shown in bold.

#### 3.2.2 Speech and language results

All 8 speech and language variables showed unadjusted associations with low vitamin B12 intake at *P* < .10, and 6 showed associations at *P* < .01 (Table 2). All the associations were consistent with the hypothesis that low vitamin B12 intake results in reduced ability in speech and language. However, allowing for the 9 potential confounders resulted in all but one outcome still showing a negative association although only 3 remained statistically significant. The one outcome that was significant at *P* < .01 concerned the intelligibility of the child's speech at 6 years of age (b = −0.28; 95% CI −0.47, −0.09; *P* = 0.005).

**Table 2:** Speech and language outcomes in children

Measure	Unadjusted			Adjusted		
	N	b [95% CI]	*P*	n	b [95% CI]	*P*
24-month vocabulary	9693	**−6.74 [10.6, −2.92]**	**<.001**	9140	**−3.98 [−7.93, −0.04]**	**.048**
38-month vocabulary	9527	**−3.09 [−5.28, −0.90]**	**.006**	8977	−0.70 [−2.96, +1.56]	.546
15-year vocabulary	4773	**−4.10 [−5.77, −2.43]**	**<.0001**	4557	−0.16 [−1.31, +0.98]	.778
38-month Word combination	9369	**−0.82 [−1.17, −0.47]**	**<.001**	8833	**−0.34 [−0.70, +0.01]**	**.054**
38-month language score	9185	**−3.51 [−6.04, −0.98]**	**.007**	8675	−1.21 [−3.82, +1.41]	.366
6-year speech intelligibility	8009	**−0.27 [−0.46, −0.09]**	**.004**	7647	**−0.28 [−0.47, −0.09]**	**.005**
WOLD comprehension	6660	**−0.21 [−0.38, −0.04]**	**.014**	6331	−0.02 [−0.19, +0.16]	.854
Pragmatic communication	7211	**−0.59 [−1.25, +0.07]**	**.078**	6892	+0.47 [−0.18, +1.13]	.157

The unadjusted and adjusted mean differences (b) between the speech and language measures for children of mothers consuming <2.26 µg/d compared to children whose mothers consumed ≥2.26 µg/d of vitamin B12 during pregnancy. Values are mean IQ points with 95% confidence intervals. Adjustments made for: mother's ethnic background; her care by her mother in childhood; her age; her current social network; her level of educational attainment; her parity 0 vs > 0 (i.e., child has older siblings); her locus of control (external vs internal), and smoking habits at 18 and 32 weeks. Results with *P* values <.10 are shown in bold.

#### 3.2.3 Mathematics and science results

The unadjusted test results for mathematics all showed strong negative associations with low prenatal intake of vitamin B12, whether using the national standardized test results or the tests devised to measure mathematical understanding (Table 3). On adjustment 2 of the 3 mathematics comprehension tests were significantly associated; in addition the third mathematics comprehension test, administered in school year 8, exhibited a very similar effect size (−0.74) to the finding in school year 6 (−0.77; 95% CI −1.33, −0.20; *P* = .008), but the latter result was based on over 6000 children compared with 2223 for school year 8.

**Table 3:** Mathematics and science outcomes in children

Test	Unadjusted			Adjusted		
	N	b [95% CI]	*P*	n	b [95% CI]	*P*
Arithmetic at 8 years	6677	**−0.51 [−0.86, −0.15]**	**.005**	6345	−0.07 [−0.43, +0.29]	.711
Math comprehension SY4 (8-9 years)	4452	**−0.72 [−1.04, −0.41]**	**<.0001**	4093	**−0.27 [−0.60, +0.05]**	**.094**
Math comprehension SY6 (10-11 years)	6740	**−1.81 [−2.36, −1.25]**	**<.0001**	6142	**−0.77 [−1.33, −0.20]**	**.008**
Math comprehension SY8 (12-13 years)	2399	**−1.79 [−2.77, −0.81]**	**<.001**	2223	−0.74 [−1.72, +0.24]	.140
Math SATS2 SY6 (10-11 years)	10245	**−4.80 [−6.17, −3.42]**	**<.0001**	9359	−0.97 [−2.33, +0.39]	.163
Math SATS3 SY9 (13-14 years)	9034	**−5.01 [−6.50, −3.51]**	**<.0001**	8215	**−2.28 [−3.81, −0.75]**	**.003**
Science reasoning	6763	**−0.24 [−0.38, −0.10]**	**<.001**	6169	+0.06 [−0.15, +0.26]	.594

The unadjusted and adjusted mean differences (b) between the mathematics and science test results for children of mothers consuming <2.26 µg/d compared to children whose mothers consumed >2.26 µg/d of vitamin B12 during pregnancy. Values are mean IQ points with 95% confidence intervals. Adjustments made for: mother's ethnic background; her care by her mother in childhood; her age; her current social network; her level of educational attainment; her parity 0 vs > 0 (i.e., child has older siblings); her locus of control (external vs internal), and smoking habits at 18 and 32 weeks. Results with *P* values <.10 are shown in bold.

Of the 2 national mathematics tests analyzed, one showed a significant association on adjustment (b = −2.28; 95% CI −3.81, −0.75; *P* = .003). Notably this was the test administered in secondary school where the mathematics was likely to be more related to reasoning than earlier national tests. There were no significant adjusted associations with measures of mental arithmetic nor with the science test results at the end of primary school (year 6).

#### 3.2.4 Reading and spelling

We analyzed the results of 7 reading tests and 2 spelling tests (Table 4). Before adjustment there were no significant associations with spelling at 7 years, comprehension at 9 years or reading speed at 9 years. There were, however, unadjusted associations with the other reading and spelling scores—in all instances the children whose mothers had low vitamin B12 intakes had lower scores. On adjustment there were no significant results with any of the reading and spelling tests.

**Table 4:** Spelling and reading outcomes in children

Test	Unadjusted			Adjusted		
	n	b [95% CI]	*P*	n	b [95% CI]	*P*
Spelling at 7 years	7115	−0.22 [−0.58, +0.15]	.238	6775	+0.07 [−0.30, +0.44]	.709
Spelling at 9 years	6875	**−0.39 [−0.71, −0.06]**	**.019**	6457	+0.03 [−0.27, +0.32]	.862
Word reading at 7 years	7221	**−0.85 [−1.61, −0.08]**	**.030**	6874	−0.16 [−0.91, +0.60]	.681
Word reading at 9 years	6875	**−0.32 [−0.56, −0.08]**	**.008**	6470	+0.06 [−0.14, +0.27]	.546
Comprehension at 9 years	6875	−0.86 [−3.73, +2.01]	.557	5868	−0.34 [−1.33, +0.66]	.505
Reading speed at 9 years	6875	+0.18 [−2.84, +3.21]	.906	5858	+0.31 [−0.78, +1.40]	.576
Reading fluency at 13 years	5004	**−1.11 [−2.12, −0.10]**	**.031**	4793	−0.23 [−1.24, +0.79]	.662
SATS3 reading score at 10-11 years	8859	**−1.29 [−1.73, −0.85]**	**<.0001**	8096	−0.34 [−0.77, +0.09]	.123
SATS3 Shakespeare reading at 13-14 years	8785	**−0.50 [−0.74, −0.25]**	**<.0001**	8008	−0.06 [−0.30, +0.19]	.646

The unadjusted and adjusted mean differences (b) between the spelling and reading test results for children of mothers consuming <2.26 µg/d compared to children whose mothers consumed ≥2.26 µg/d of vitamin B12 during pregnancy. Values are mean IQ points with 95% confidence intervals. Adjustments made for: mother's ethnic background; her care by her mother in childhood; her age; her current social network; her level of educational attainment; her parity 0 vs > 0 (i.e., child has older siblings); her locus of control (external vs internal), and smoking habits at 18 and 32 weeks. Results with *P* values <.10 are shown in bold.

## 4 Discussion

In this study, we have investigated whether there is evidence for a prenatal diet low in vitamin B12 being associated with the cognitive development of the child beyond the age of 3 years. We have assessed the child's cognitive development using a variety of sources including: maternal responses to specific questions (for speech and language), direct tests performed by the ALSPAC team (reading and spelling), specific tests designed to assess the mathematical and scientific comprehension of the child and results of national tests. We have demonstrated that, once the potential confounders were taken into account, there was evidence of adverse associations with specific aspects of speech and of mathematics comprehension. An earlier analysis of ALSPAC data only assessed one outcome (total IQ) and used the dietary intake as continuous [50] and did not assess adequacy of intake; they showed a marginal association, which was supported by a Mendelian Randomization study.

Vitamin B12 is naturally found in animal products, including fish, meat, poultry, eggs, milk, and milk products. It is generally not present in plant foods, but fortified breakfast cereals are a readily available source of the vitamin. Consequently, the group of women who have a low intake of B12 may have had a low intake of animal protein and a failure to eat fortified cereals or yeast extract products. At a time when vegetarianism and veganism are both becoming more popular it is important that women avoiding meat and fish in pregnancy are made aware of the possibilities of their diet being inadequate in nutrients such as vitamin B12.

We have shown in the Supplemental material that the women who had a low dietary intake were likely to be from a nonwhite ethnic background, have poor care from their own mother in childhood, a poor social network, lower educational attainment, were expecting their first child (parity 0), were more likely to smoke and to smoke heavily, and to have an external locus of control. Taking these factors into account reduced the strength of the associations with most cognitive outcomes. It is noteworthy that among the few (6) adjusted results that remained as statistically significant, there was a clear message in regard to specific outcomes concerning mathematics comprehension as opposed to mental arithmetic.

The tests of mathematics comprehension used by the study aimed to assess the level of children's reasoning about quantities and about the relationships between quantities in mathematical problems independently of their computation skills. None of the items in these tests contained difficult calculations; the children had to reflect on the relationships between quantities in each item to decide how to solve the problem (see Section 2.5.6.1). Thus, the tests relate to concepts different from the computational skills tested by mental arithmetic. This raises the hypothesis that vitamin B12 has an effect on the brain that influences the centers that are responsible for the type of logical processing exhibited by these tests. No other studies have specifically found associations of maternal vitamin B12 and children's mathematics comprehension, but this could in part be due to the limited measurements/tests administered and the young ages of most assessments [17,^19^,21].

Results concerning speech and language were less clear. Two of these concerned early vocabulary and word combination scores and were of borderline significance at the traditional 5% level. The other factor concerned the intelligibility of speech. This scale combined the results from a series of questions completed by the mother concerning how well the child was understood by parents, other members of the family and visitors to the home. The results indicate that the children born to mothers who had a low intake of B12 were less likely to be understood. Adjustment for a variety of factors made little difference to the association. This is supported by one observational study [17] on maternal B12 intake from food/supplements during the second trimester which showed an association with children's receptive language at age 3, but this association was not seen in these children at age 7 [18]. However, this is likely to have been affected by high levels of attrition by this age. The one randomized controlled trial of maternal vitamin B12 and offspring cognition also showed elevated maternal tHcy levels to be associated with poorer cognitive performance in the domain of expressive language [19]. No association was seen of B12 supplementation directly, but this may have been due to the limited timing of dosage, and young age of assessment (9 months old) [19]. Additionally, in a study in rural India of children at 9 to 10 years of age, a measure of verbal fluency was the only positive association found between maternal vitamin B12 status and cognitive performance [7, 52]. Together these results corroborate our findings to some extent.

### 4.1 Strengths and limitations

There are a number of limitations to this study. Although we have shown some adverse cognitive associations with reduced intake of vitamin B12, we only considered low intakes as identified by the lowest 10% of intake of the pregnant woman—there may be more dramatic associations with the much lower intakes found in countries such as India for example [17,53]. We have no biological measures of plasma or serum levels of vitamin B12 to test the validity of the dietary measure as an assessment of vitamin B12 status. There is the possibility of mother's misreporting dietary information in the questionnaire, and additionally different foods can yield different blood concentrations of vitamin B12 [50]. Although we used a comprehensive exposome set of analyses to determine the confounders to be used in this study, it may be that there are other factors that should have been taken into account. The measure of diet in pregnancy was administered at 32 weeks gestation and asked about foods consumed “nowadays.” Consequently, it is unable to answer questions relevant to early pregnancy—the time at which nausea is most prevalent with consequent dietary change (however, it is thought that there are sufficient stores of vitamin B12 in the liver to prevent any women other than those who were already very deficient to show a deficit at this time). There is evidence to suggest that the timing of vitamin B12 intake is important; nutrient deficiency may be more likely to impair brain development during a time period when the need is specifically high for that nutrient [17,51]. We were unable to trace any longitudinal study that included tests of mathematics comprehension that could be used to validate our key findings. Although the study is likely to be representative of the UK population in the 1990s, it is unclear whether it can be considered generalizable to persons from other cultures and genetic backgrounds, or to more current diets. It is likely that mothers with a diet low in vitamin B12 are likely to have a low intake of other nutrients present in similar foods to B12. Several other nutrients in pregnancy have been found to have associations with cognitive outcomes in children. Two out of 3 studies of vitamin D have shown low cognitive ability in children of deficient mothers, although there is a lack of evidence from developing countries or controlled trials [1]. It has been hypothesized that folic acid causes significant changes in DNA methylation in cord blood of genes related to brain development; studies of maternal intake on the effect of offspring cognition have found mixed results, but some evidence of an association (including with supplementation) [1,54]. As indicated in the introduction, even a mild to moderate deficiency of iodine may be associated with subtle impairments in cognition and school performance [1,55]. Thus, residual confounding of multiple nutrients and their interactions is a concern in our study and many other observational studies [1].

Among the strengths of the study are: (1) the fact that the study is based on a population of pregnant women defined by a geographic area of residence, and consequently unlikely to be biased by selection; follow-up of the children comprised relatively large numbers; and follow-up tests used a variety of methods including linkage to national test results which were not biased by the fact that certain social groups were more likely to attend for testing at the ALSPAC premises. In addition, whether or not the child was tested did not depend on whether the mother's diet was high or low in vitamin B12.

Given the overall mixed results from the literature on the long-term associations between low prenatal vitamin B12 intake and the child's cognitive outcomes, the results from this study specific to mathematical comprehension must be considered to be hypothesis generating rather than a definitive answer to the overall study hypothesis. We now hypothesize that if our association with mathematical comprehension is true, then this will be likely to be supported by equivalent gene-environment association analyses in the future.

## Appendix Supplementary materials

Image, application 1
