# Supplementary material for: Maternal prenatal vitamin B12 intake is associated with speech development and mathematical abilities in childhood
Source: Nutr Res. 2021 Feb;86:68–78. doi: 10.1016/j.nutres.2020.12.005 (PMC7870459; doi:10.1016/j.nutres.2020.12.005)
Supplement: Supplementary file 1 [file mmc1.docx]

**Supplemental Materials: Maternal prenatal vitamin B12 intake is associated with speech development and mathematical abilities in childhood**

**Supplemental Table S1. The Exposome of variables related to the mothers’ childhood and their associations with deficiency in vitamin B12 consumption:**

| **Study mothers’ childhood** | **Univariable analyses** | | | **Multivariable analysis** | |
| --- | --- | --- | --- | --- | --- |
|  | **N** | **OR [95% CI]** | **P** | **OR [95% CI]** | **P** |
| Ethnic background is white | 11944 | 0.479 [0.356, 0.644] | **1.1 x 10^-6^** | 0.584 [0.421, 0.811] | **0.001** |
| Number of her older siblings | 12082 | 1.109 [1.056, 1.165] | **3.8 x 10^-5^** | 1.062 [1.007, 1.121] | **0.026** |
| Number of her younger siblings | 12079 | 1.108 [1.053, 1.165] | **7.3 x 10^-5^** | 1.069 [1.013, 1.129] | **0.016** |
| Her mother was present in household when aged ≤5 | 11533 | 0.606 [0.478, 0.769] | **3.7 x 10^-5^** |  |  |
| Very happy when aged ≤5 | 10023 | 0.744 [0.632, 0.875] | **3.7 x 10^-4^** |  |  |
| Her mother was present in household when aged 6-11 | 11533 | 0.594 [0.483, 0.731] | **8.6 x 10^-7^** |  |  |
| Her father present in household when aged 6-11 | 11533 | 0.666 [0.566, 0.784] | **1.0 x 10^-6^** |  |  |
| Very happy when aged 6-11 | 11294 | 0.792 [0.696, 0.900] | **3.7 x 10^-4^** |  |  |
| Her mother present in household when aged 12-15 | 11533 | 0.595 [0.482, 0.735] | **1.4 x 10^-6^** | 0.749 [0.596, 0.942] | **0.013** |
| Her father present in household when aged 12-15 | 11533 | 0.677 [0.584, 0.784] | **2.0 x 10^-7^** | 0.844 [0.718, 0.992] | **0.040** |
| Her mother’s level of care towards her | 11532 | 0.978 [0.968, 0.989] | **4.5 x 10^-5^** | 0.982 [0.971, 0.993] | **0.001** |
| Her age at the time of pregnancy | 12095 | 0.950 [0.938, 0.962] | **6.1 x 10^-16^** | 0.957 [0.945, 0.970] | **1.5 x 10^-10^** |

The multivariable analyses give the mutually adjusted results of backwards stepwise analyses with 95% confidence intervals N=11397, R2=1.57%

**Supplemental Table S2. The Exposome of variables related to the mothers’ socioeconomic status at the time of pregnancy and their associations with deficiency in vitamin B12 consumption.**

| **Socio-economic status at time of** | **Univariable analyses** | | | **Multivariable analysis** | |
| --- | --- | --- | --- | --- | --- |
| **Pregnancy** | **N** | **OR [95% CI]** | **P** | **OR [95% CI]** | **P** |
| Home is owned/mortgaged | 11715 | 0.577 [0.507, 0.657] | **1.0 x 10^-16^** | 0.781 [0.675, 0.905] | **0.001** |
| Home is centrally heated | 11776 | 0.690 [0.588, 0.810] | **5.8 x 10^-6^** |  |  |
| Dissatisfaction with home | 11734 | 1.260 [1.165, 1.364] | **8.6 x 10^-9^** |  |  |
| Lives with partner | 11472 | 0.642 [0.509, 0.810] | **1.8 x 10^-4^** |  |  |
| Crowding index | 11544 | 1.367 [1.242, 1.504] | **1.7 x 10^-10^** |  |  |
| Social network score | 11494 | 0.937 [0.923, 0.951] | **4.5 x 10^-18^** | 0.957 [0.942, 0.972] | **6.3 x 10^-8^** |
| Her education level | 12025 | 0.758 [0.724, 0.795] | **7.0 x 10^-31^** | 0.814 [0.772, 0.858] | **2.5 x 10^-14^** |
| Partner’s education level | 11570 | 0.826 [0.792, 0.862] | **6.1 x 10^-19^** |  |  |
| Partner’s social class | 10915 | 1.134 [1.080, 1.191] | **4.8 x 10^-7^** |  |  |
| Financial difficulties score | 12032 | 1.031 [1.015, 1.048] | **1.8 x 10^-4^** |  |  |

N=11174, Pseudo R^2^=2.20%

The multivariable analyses give the mutually adjusted results of backwards stepwise analyses with 95% confidence intervals

**Supplemental Table S3.** Combination of independent variables from Supplemental Tables S1 and S2.

|  | **Univariable analyses** | | | **Multivariable analysis** | |
| --- | --- | --- | --- | --- | --- |
|  | **N** | **OR [95% CI]** | **P** | **OR [95% CI]** | **P** |
| Ethnic background is white | 11944 | 0.479 [0.356, 0.644] | **1.1 x 10^-6^** | 0.519 [0.374, 0.720] | **8.5 x 10^-5^** |
| Number of her older siblings | 12082 | 1.109 [1.056, 1.165] | **3.8 x 10^-5^** |  |  |
| Number of her younger siblings | 12079 | 1.108 [1.053, 1.165] | **7.3 x 10^-5^** |  |  |
| Her mother was present in household when aged 12-15 | 11533 | 0.595 [0.482, 0.735] | **1.4 x 10^-6^** |  |  |
| Her father was present in household when aged 12-15 | 11533 | 0.677 [0.584, 0.784] | **2.0 x 10^-7^** |  |  |
| Her mother’s care of her score | 11532 | 0.978 [0.968, 0.989] | **4.5 x 10^-5^** | 0.986 [0.975, 0.997] | **0.016** |
| Her age at the time of pregnancy | 12095 | 0.950 [0.938, 0.962] | **6.1 x 10^-16^** | 0.970 [0.957, 0.984] | **1.4 x 10^-5^** |
| Home is owned/mortgaged | 11715 | 0.577 [0.507, 0.657] | **1.0 x 10^-16^** |  |  |
| Social network score | 11494 | 0.937 [0.923, 0.951] | **4.5 x 10^-18^** | 0.960 [0.944, 0.975] | **6.9 x 10^-7^** |
| Her education level | 12025 | 0.758 [0.724, 0.795] | **7.0 x 10^-31^** | 0.820 [0.778, 0.864] | **1.9 x 10^-13^** |

N=11318, Pseudo R^2^=2.53%

The multivariable analyses give the mutually adjusted results of backwards stepwise analyses with 95% confidence intervals

**Supplemental Table S4.** **The Exposome of variables related to the mothers’ childhood and their associations with deficiency in vitamin B12 consumption.**

| **Lifestyle in pregnancy** | **Univariable analyses** | | | **Multivariable analysis** | |
| --- | --- | --- | --- | --- | --- |
|  | **N** | **OR [95% CI]** | **P** | **OR [95% CI]** | **P** |
| Parity >0 | 11653 | 0.793 [0.702, 0.896] | **1.9 x 10^-4^** | 0.766 [0.673, 0.872] | **5.6 x 10^-5^** |
| Smoked mid-pregnancy | 11852 | 1.195 [1.029, 1.387] | **0.019** | 0.790 [0.634, 0.984] | **0.035** |
| Amount smoked at 32 weeks gestation | 12095 | 1.403 [1.250, 1.574] | **9.3 x 10^-9^** | 1.375 [1.149, 1.645] | **0.001** |
| No passive smoke exposure | 10487 | 0.725 [0.632, 0.831] | **4.2 x 10^-6^** |  |  |
| No alcohol consumed mid-pregnancy | 11533 | 1.352 [1.194, 1.530] | **1.9 x 10^-6^** | 1.263 [1.109, 1.438] | **4.2 x 10^-4^** |
| Locus of control score of the mother | 11533 | 1.156 [1.123, 1.190] | **1.0 x 10^-22^** | 1.155 [1.120, 1.192] | **7.7 x 10^-20^** |

N=10925, Pseudo R^2^=1.97%

The multivariable analyses give the mutually adjusted results of backwards stepwise analyses with 95% confidence intervals

**Supplemental Table S5.** **Combination of independent variables from Supplemental Tables S3 and S4.**

| **Features of study mother before and during pregnancy** | **Univariable analyses** | | | **Multivariable analysis** | |
| --- | --- | --- | --- | --- | --- |
|  | **N** | **OR [95% CI]** | **P** | **OR [95% CI]** | **P** |
| Ethnic background is white | 11944 | 0.479 [0.356, 0.644] | **1.1 x 10^-6^** | 0.507 [0.363, 0.707] | **6.4 x 10^-5^** |
| Her mother’s care of her score | 11532 | 0.978 [0.968, 0.989] | **4.5 x 10^-5^** | 0.988 [0.977, 0.9999] | **0.049** |
| Her age | 12095 | 0.950 [0.938, 0.962] | **6.1 x 10^-16^** | 0.982 [0.968, 0.997] | **0.018** |
| Social network score | 11494 | 0.937 [0.923, 0.951] | **4.5 x 10^-18^** | 0.963 [0.947, 0.979] | **1.2 x 10^-5^** |
| Her education level | 12025 | 0.758 [0.724, 0.795] | **7.0 x 10^-31^** | 0.836 [0.789, 0.887] | **2.7 x 10^-9^** |
| Parity >0 | 11653 | 0.793 [0.702, 0.896] | **1.9 x 10^-4^** | 0.746 [0.650, 0.857] | **3.4 x 10^-5^** |
| Smoked mid-pregnancy | 11852 | 1.195 [1.029, 1.387] | **0.019** | 0.716 [0.574, 0.893] | **0.003** |
| Amount smoked at 32 weeks gestation | 12095 | 1.403 [1.250, 1.574] | **9.3 x 10^-9^** | 1.320 [1.104, 1.580] | **0.002** |
| No alcohol consumed mid-pregnancy | 11533 | 1.352 [1.194, 1.530] | **1.9 x 10^-6^** |  |  |
| Locus of control score | 11533 | 1.156 [1.123, 1.190] | **1.0 x 10^-22^** | 1.071 [1.034, 1.109] | **1.5 x 10^-4^** |

N=11012, Pseudo R^2^=3.14%

The multivariable analyses give the mutually adjusted results of backwards stepwise analyses with 95% confidence intervals
